# Paediatric out-of-hospital resuscitation in an area with scattered population (Galicia-Spain)

**DOI:** 10.1186/1471-227X-7-3

**Published:** 2007-05-14

**Authors:** Pilar Blanco-Ons Fernández, Luis Sánchez-Santos, Antonio Rodríguez-Núñez, José Antonio Iglesias-Vázquez, María Cegarra-García, Maria Victoria Barreiro-Díaz

**Affiliations:** 1 Melide's Primary Care Center, Servicio Galego de Saúde (SERGAS), Melide, Coruña, Spain; 2 Arzúa's Primary Care Center, Servicio Galego de Saúde (SERGAS), Arzúa, Coruña, Spain; 3 Pediatric Emergency and Critical Care Division, Department of Pediatrics, Hospital Clínico Universitario de Santiago de Compostela, Servicio Galego de Saúde (SERGAS) and Universidad de Santiago de Compostela, Santiago de Compostela, Spain; 4 Public Foundation Emergency Medical System of Galicia-061, Servicio Galego de Saúde (SERGAS), Santiago de Compostela, Spain

## Abstract

**Background:**

Cardiorespiratory arrest (CRA) is a rare event in childhood. Our objective was to determine the characteristics of paediatric CRA and the immediate results of cardiopulmonary resuscitation (CPR) in Galicia, a community with a very scattered population.

**Methods:**

All children (aged from newborn to 16 years old) who suffered an out-of-hospital CRA in Galicia and were assisted by the Public Foundation Medical Emergencies of Galicia-061 staff, from June 2002 to February 2005, were included in the study. Data were prospectively recorded following the Utstein's style guidelines.

**Results:**

Thirty-one cases were analyzed (3.4 CRA annual cases per 100.000 paediatric population). The arrest was respiratory in 16.1% and cardiac in 83.9% of cases. CRA occurred at home in 58.1% of instances. Time CRA to initiation of CPR was shorter than 10 minutes in 32.2% and longer than 20 minutes in 29.0% of cases. 22.6% of children received bystander CPR. The first recorded rhythm was asystole in 67.7% of cases. Bag-mask ventilation was used in 67.7% and in 83.8% oro-tracheal intubation was done. A peripheral venous access was achieved in 67.7% and intraosseous access was used in 16.1% of patients. 93.5% of children were treated with adrenaline. After initial CPR, sustained restoration of spontaneous circulation was achieved in 38.7% of cases. Six children (19.4%) survived until hospital discharge. Four of 5 children with respiratory arrest survived, whereas only 2 of 26 children with cardiac arrest survived until hospital discharge.

**Conclusion:**

Despite the handicap of a highly disseminated population, paediatric CRA characteristics and CPR results in Galicia are comparable to references from other communities. Programs to increase bystander CPR, equip laypeople with basic CPR skills and to update life support knowledge of health staff are needed to improve outcomes.

## Background

Cardiorespiratory arrest (CRA) is a rare event during childhood that needs early and adequate treatment in order to achieve survival without neurological damage [1-6]. In addition to previous retrospective data, recent studies have increased our knowledge about characteristics and prognosis of CRA in children [1-7]. The Pediatric Utstein style has facilitated systematic recording and evaluation of data and permitted the comparison of results between different groups and regions [8]. Most studies about out-of-hospital paediatric CRA have been done in mainly urban areas; this factor could have influence on the results of cardiopulmonary resuscitation (CPR) and might not be extrapolated to predominantly rural areas or regions with highly dispersed population. A previous multicenter study of out-of-hospital CRA in Spain found that survival was higher when resuscitation was started soon by laypersons or paramedics, the neurological outcome of survivors was quite good (81.% had scores 1 or 2 in the Pediatric Cerebral Performance Scale at hospital discharge) and the best indicator of mortality was duration of resuscitation efforts [1].

The objective of the present study was to determine the characteristics of paediatric CRA and the immediate results of CPR in a Spanish region characterized by its scattered population.

## Methods

Galicia is an autonomous region in Spain located at the Northwest of Iberian Peninsula, with an area of 29.575 Km^2^. According to 2000's census, it has 2.732.926 inhabitants (6.7% of total Spain's population) in a territory that has 5.8% of Spain surface. Galicia's population distribution is characterized by an extreme dispersion, indicated by the fact that it accounts for 51.6% of all the Spain's population sites and that 54.2% of population lives in towns with less than 20.000 inhabitants [9]. Galicia has 99 villages per 100 Km^2 ^(12 in Spain) with a mean of 94 inhabitants per village (639 in Spain) [9]. Demographics of Galicia is characterized by a regressive population's pyramid, with a high percentage of old people (in year 2005, 19% were older than 65 years and only 13% were younger than 15 years). Ethnicity is very homogeneous with a very low percentage of immigrants. Economic indicators of Galicia indicate that our community is below the mean of other regions in Spain as well as other Economic European Community countries [9].

The Emergency Medical System in Galicia is a public institution that included, during the study period, a medically dispatch coordinating centre, two advanced life support (ALS) helicopters (with one emergency doctor and one nurse as crew), 8 ALS ambulances (with one emergency doctor and one nurse as crew) located in main cities and a network of 93 ambulances crewed by two paramedics with ability to carry out basic life support, bag and mask ventilation and automated external defibrillation.

This system has been designed to assure arrival of ALS for 90% of population and BLS for 100% in less than 20 minutes. All medical emergency calls in Galicia go to a Medical Emergency Coordination Centre (MECC) 24 hours of the day. In less than three minutes, the Centre's staff must decide the most adequate resource for each specific situation. The helicopter's and ambulance's crew are in permanent contact with the MECC. BLS ambulances must follow the doctor in charge indications. Helicopters are operative only in daylight hours. Ambulances operate around the clock. ALS ambulances cover mainly urban areas and BLS ambulances are distributed to cover both urban and rural areas.

BLS ambulance staff has passed a formation program that includes courses on basic and immediate life support, automated external defibrillation, pre-hospital trauma care and transport. A retraining course is required at least every year. ALS ambulance and helicopter staff (physician and nurses) must have specific training in emergencies and passed at least five courses: ALS, PLS, Pre-hospital trauma care, Transport and Emergency Coordination. The EMS in Galicia has created a Teaching Centre to fulfil its specific training needs. Life support courses and treatment protocols follow the recommendations of the European Resuscitation Council.

The EMS is coordinated with the hospital system. In our country, there is a Public Health System that covers all population and includes a stratified hospital network. Patients assisted by the EMS, if deemed, are transported to the most adequate hospital, being the MECC doctor in charge the responsible for the decision.

In the present study we have collected all the children aged from newborn to 16 years-old that suffered a CRA at out-of-hospital level and were immediately or subsequently assisted by the Galicia's EMS staff. Recruitment period included 32 months (from June 2002 to February 2005). In each case, the person in charge fulfilled a case report sheet and given a verbal report to the coordinating centre. Data were prospectively included in a standardized database designed following the international paediatric Utstein style recommendations [8]. Information about survival to hospital discharge was obtained from the Paediatric Intensive Care Units' staff in each case.

Respiratory arrest (RA) was defined as the absence of respiration requiring assisted ventilation. Cardiac arrest (CA) was defined as the inability to palpate a central pulse, unresponsiveness and apnoea or severe bradycardia lower than 60 bpm with poor perfusion in infants requiring external cardiac compressions and assisted ventilation [1,2,8].

Analyzed data included patient-related variables: age, cause of the arrest, and personal background); arrest and life support related variables: type of arrest, location of the arrest, time elapsed from the arrest to starting of cardiopulmonary resuscitation (CPR), persons who performed the CPR life support manoeuvres, first documented ECG rhythm, and total duration of CPR); and outcome-related variables: return of spontaneous circulation and survival to hospital discharge.

### Statitstical methods

Data analysis was performed with StatgraphicsPlus 5.1 statistical package. Due to the number of patients, only descriptive statistics (number, percentage, median, range) are reported. The study was approved by the EMS of Galicia ethics board.

## Results

During the study period 31 children suffered from an out-of-hospital CRA and were assisted by the Galicia's EMS, that means 3.4 cases per 100.000 children and year. Patients' age ranged from newborn to 15 years (median: 5 years).

Type of arrest, suspected arrest's cause, place where arrest occurred and estimated time elapsed from arrest to start of CPR and response times of EMS for these patients are showed in table 1.

**Table 1:** Characteristics of paediatric cardiorespiratory arrest in Galicia.

	N	%
Type of arrest		
- Respiratory	5	16.1
- Cardiac	26	83.9
Suspected etiology		
- Respiratory diseases	8	25.8
- Heart diseases	5	16.1
- Trauma	5	16.1
- Drowning	5	16.1
- Intoxication	2	6.4
- Other	6	19.3
Place of arrest		
- Public place	13	41.9
- At home	18	58.1
Estimated time elapsed from arrest to start of CPR		
- < 4 min	4	12.9
- 4 – 10 min	6	19.3
- 10 – 20 min	10	32.2
- > 20 min	9	29.0
- unknown	2	6.4
EMS response time		
- < 4 min	2	6.4
- 4 – 10 min	9	29.0
- 10 – 20 min	11	35.5
- > 20 min	9	29.0

Seven children (22.6%) received bystander CPR. In two cases (6.4%), CPR was started in a primary care facility and in other two (6.4%) it was initiated by ambulance paramedics. In 27 cases (87.1%), first health assistance was done by the ALS ambulance staff. First detected rhythm was asystole in 21 cases (67.7%); two cases (6.4%) presented a shockable rhythm and other rhythms were documented in the remaining 8 (25.8%). CPR procedures applied in theses patients are showed in table 2.

**Table 2:** Cardiopulmonary resuscitation procedures and treatments.

	N	%
Airway control		
- Bag and mask	3	9.7
- Bag-mask and tracheal intubation	18	58.1
- Immediate tracheal intubation	8	25.8
Vascular access		
- Peripheral intravenous	21	67.7
- Intraosseous	5	16.1
- Tracheal route	5	16.1
- Central venous	3	9.7
- Umbilical	1	3.2
Adrenaline	29	93.5
- One dose	11	35.5
- Two doses	5	16.1
- Three or more doses	13	41.9
Atropine	17	54.8
Bicarbonate	4	12.9
Fluids	10	32.2
Defibrillation	2	6.4

**Figure 1:**
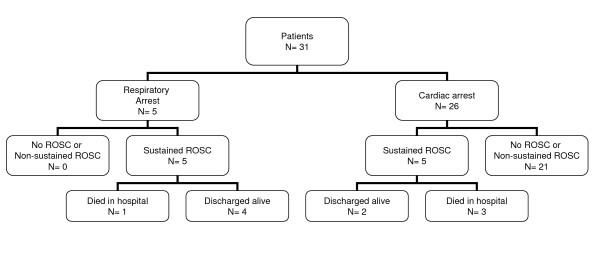
**Evolution of patients**. (ROSC: Return of spontaneous circulation).

Outcome of patients is shown in Figure 1. Four of 5 children with RA were discharged alive, that compares with 2 of 26 with CA.

## Discussion

Out-of-hospital paediatric CRA has poor outcome [6], but immediate and coordinated treatment according to the "chain of survival" and international guidelines should contribute to improve this situation [11,12]. In order to check performance quality of individual emergency medical systems, each local institution should review its results and try to make comparison with international counterparts.

Our study, although limited by the number of cases, gives insight on the results of paediatric out-of-hospital CPR in a region with special demographics that challenge the EMS performance. Despite the wide dispersion of our population settlement (with less than 7% of Spain's population, Galicia has more than half of population's places) our data are similar to others, at national [1,2] and international level [5-7,10].

Out-of-hospital paediatric CRA incidence is not well known; several studies from different areas have reported figures ranging from 2.6 to 19.7 yearly cases per 100.000 children [3,4,6,13]. Our incidence is located in the low part of this range. Although it can be argued that a number of patients might have been resuscitated only at their arrival to an hospital emergency department, these hypothetical cases, if any, must be very few. The structured and public organisation of medical emergencies in Galicia nearly permits to discard the possibility that an out-of-hospital CA victim arrives to hospital without prior assistance.

The place where arrest occurs may be an important prognostic factor, because it determines the time elapsed from CRA to resuscitation manoeuvres [1,6]. When compared with a previous study in Spain, in Galicia nearly twice as many events occurred at home. This fact may explain, at least in part, the long time elapsed from arrest to the start of CPR in our series: only 32.3% of cases were resuscitated in the first 10 minutes, figure that compares with 62% in the study by Lopez-Herce et al. [1].

A main prognostic factor of CRA is type of arrest, with respiratory arrest having more favourable outcome than cardiac arrest [1,2,6]. In this sense, it is remarkable that 84% of our cases had cardiac arrest at the time when CPR was started. This fact could indicate that in most instances, resuscitation procedures were initiated later in time, and it underlines the paramount importance of recognition of seriously ill children, as well as the need for early bystander and sanitary staff resuscitation attempts [14,15]. It is noteworthy that in our series bystander resuscitation occurred only in 22.6% of cases, a figure clearly lower than international reports [6] (Table 3). Therefore, we consider essential the implementation of local strategies with the objective that all citizens know and perform immediately BLS procedures [16]. We hope that current (2005) CPR universal algorithms will contribute to increase the rate of bystander CPR, independently of the victim's age [14-17].

**Table 3:** Comparison of our results with other, at national and international level.

	Present study	López-Herce et al (1)	Donoghue et al (6)
Location	Galicia	Spain	International
Study characteristics		Multicentre	Metanalysis
Bystander CPR	22.6	15.9	30.7
First documented rhythm			
- Asystole	67.7	64.2	78.0
- VF/PVT	6.4	9.5	8.0
CPR outcome			
- Sustained ROSC	38.7	47.3	27.8
- Survival to hospital admission	32.2		23.9
- Survival to hospital discharge	19.4	28.0	12.1

Results in percent.

CPR: Cardiopulmonary resuscitation

VF/PVT: Ventricular fibrillation/Pulseless ventricular tachycardia

ROSC: Return of spontaneous circulation

Asystole is the most frequently first documented rhythm in all pediatric CRA series [1,5,6]. This fact has been related with the specific hypoxic patho-physiology of cardiac arrest in children and also with a relatively late assistance of paediatric victims [6]. Although shockable rhythms are not frequent in paediatric series, its incidence in our population has been very rare (Table 2). One possible explanation could be the long time elapsed between arrest and the start of ECG monitoring.

Although the resuscitation procedures were similar to those reported by Lopez-Herce et al. [1], in our series it is remarkable the high percentage of tracheal intubation (84%), that could indicate a high level of skill of our sanitary staff. On the other hand, intraosseous access was used only in 16% of cases, a result that compares with 29% in the aforementioned study [1]. We consider that this fact is not related with ignorance of the technique (all our physicians and nurses have passed a Paediatric Advanced Life Support course that includes an intraosseous access skillstation) but may be explained by a relatively high success with peripheral venous access (67%).

It is noteworthy that 55% of patients were treated with atropine, an essential drug in adult's CPR algorithms but clearly secondary in paediatric ones. In our opinion, this attitude could be a consequence of the adult's medicine background of our emergency health staff and the predominance of CRA adult victims. In contrast, bicarbonate was administered in few instances (13% vs. 44% in the Spanish study) [1], without a clue that could help us to explain this fact.

In order to assess the results of CPR, survival rates are essential but comparison between different EMS must be cautious because of differences in populations, EMS organisation and number of arrest included in each study. We have obtained lower figures than those reported by Lopez-Herce et al. [1] but higher than those reported by Donoghue et al. [6] (Table 3). Results are not easy to compare, because the interval CRA-CPR was longer in our series than other studies and also because the international epidemiological review [6] includes several studies published before 1995 and also retrospective analysis, that resulted in a wide range of survival to hospital discharge (from 0 to 31%).

Our study has some limitations. Our population and CRA incidence are low to permit detailed and reliable statistical analysis of response to CPR and survival factors. Due to the low incidence of arrest in children, only multicentre and/or prolonged in time studies make possible such assessments, with significant risks of bias due to inter-areas variability and to changes in treatment guidelines as well as resources along time. Also, the amount and quality of information that can be obtained from out-of-hospital CRA is limited because of the lack of time to obtain data and the uncontrolled scenario.

In addition, having in mind that the true objective of CPR is not crude survival but restoration of vital functions without neurological damage [1,6], the main focus of outcome studies should be to know the long-term neurological status of survivors. In our case, these data are not available because a follow-up of patients after hospital admission was not done. In this sense, we are convinced that the assessment of neurological status of survivors one year after the event, should be essential in the design of future studies on the subject [1,2].

## Conclusion

Our results suggest that paediatric CRA characteristics and CPR results in Galicia are comparable to those obtained in other areas at national and international scale, although specific aspects derived from our community circumstances, are present. However, available data are limited and must be confirmed by prospective and more detailed studies on the subject, including long term outcome assessment. We consider that programs with the objective to improve bystander CPR rate, increase BLS knowledge and predisposition to resuscitate by laypeople, as well as to update paediatric life support knowledge and skill of health staff should be implemented in our community.

## Competing interests

The author(s) declare that they have no competing interests.

## Authors' contributions

PBOF and LSS analyzed data and drafted the manuscript. ARN conceived the study, participated in the design of the study and drafted the manuscript. JAIV participated in the design, coordinated the study and reviewed the manuscript. MCG and MVBD participated in the design and reviewed the manuscript. All authors read and approved the final manuscript.

## Pre-publication history

The pre-publication history for this paper can be accessed here:


